# Bats data from fragmented forests in Terengganu State, Malaysia

**DOI:** 10.1016/j.dib.2020.105567

**Published:** 2020-04-22

**Authors:** Nurulhuda Zakaria, Athirah Ahmad Tarmizi, Muhammad Alif Mat Zuki, Amirrudin Bin Ahmad, Mazrul Aswady Mamat, Mohd Tajuddin Abdullah

**Affiliations:** a Faculty of Science and Marine Environment, Universiti Malaysia Terengganu, 21030 Kuala Nerus, Terengganu, Malaysia; b Institute of Tropical Biodiversity and Sustainable Development, Universiti Malaysia Terengganu, 21030 Kuala Nerus, Terengganu, Malaysia

**Keywords:** Chiropteran, Logged forest, Oil palm plantation, Riparian, Body condition index, Population structure

## Abstract

This data article is about bats observed from fragmented forest understories interspaced by agricultural plantations, utility corridors, and man-made structures within rural areas of Setiu (Bukit Kesing Forest Reserve and Ladang Tayor TDM) and Hulu Terengganu (Pengkalan Utama and Sungai Buweh, Kenyir) that are situated in Terengganu state, Peninsular Malaysia. Surveys were conducted from October 2018 until January 2019. These bats were captured using harp traps and mist nets that were set 30 m apart across flyways, streams, rivers and less cluttered trees in the 50 m transect zones (identified at each site). All animals captured were distinguished by morphology and released at the same location it was caught. The data comprise of 15 species of bats from four family groups, namely Hipposideridae, Pteropodidae, Rhinolophidae and Vespertilionidae. The data were interpreted into weight-forearm length (W-FA) to inform about bats Body Condition Index (-0.25 to 0.25).

Specifications table

**Table:** 

Subject	Biology
Specific subject area	Bioscience and Biodiversity
Type of data	Tables
How data were acquired	Four bank harp trap (4.2 m^2^) and mist nets (height = 2.6 m and width = 10 m), Vernier caliper (sensitivity 0.1 cm), measuring tape and analytical balance (sensitivity 0.1 kg)
Data format	Raw Semi-analyzed
Parameters for data collection	Harp trap and net placement at flyways, streams and less cluttered trees and, the 10 m spacing interval between the traps and nets.
Description of data collection	Descriptive abundance, age structure, sex, guild type, trapping method and weight to length (W-L) relationships were used to describe bats from Setiu and Hulu Terengganu districts. The species recorded list was compared to International Union for Conservation of Nature Red List (IUCN Red List) database to determine the conservation status of species.
Data source location	1. Setiu District, Terengganu, East Peninsular Malaysia Bukit Kesing Forest Reserve: N 5.262510°, E 102.873038° Ladang Tayor TDM: N 5.253675°, E 102.883917° 2. Hulu Terengganu District, Terengganu, East Peninsular Malaysia Pengkalan Utama, Kenyir: N 5.142650°; E 102.760355° Sungai Buweh, Kenyir: N 5.147635°, E 102.768261°
Data accessibility	All raw data are available within this article

## Value of the data

• Data on abundance and distribution of bats are beneficial for the scientific community to understand diversity patterns and spatial distribution of bats species within their habitat which is important for interpreting ecological processes as bats such as pollinator of orchard and agricultural plantation crops, dispersal agent of seeds, and natural biological control predator of insect pests.
• Data on bat species from different guilds is necessary for scientific community to investigate the effects of monoculture agriculture on abundance, diversity, and foraging behaviour of frugivorous bats.
• Sex and life stage data will allow scientific community to determine key reproductive features such as timing of reproductive activity, and to determine seasonal patterns in body masses of adult males and females in relation to energetic costs of different stages of the life cycle.
• Body condition index that indicate individual fitness of bats could be extended towards predicting reproductive traits and survival of bats, resource acquisition and allocation, nutritional status, immune-competence and stress.
• Data on different methods of sampling bats can give an opportunity to scientific community to assess the variation among species of bats in their susceptibility to traps.
• Comparative abundance of bats in fragmented and non-fragmented habitats, scientific community can explore further about the variety of bats present in agricultural, rural s, inhabited and forested areas and examine the effects of landscape changes on bats. Such information is important and useful for the authorities for planning and implementation of species conservation and management.

## 1 Data

The dataset in this article is constructed using field survey results that indicate abundance of bats in fragmented forests within Terengganu state with all captured bats were identified, and enumerated. Table 1 describes the abundance of bats, number of species, number of field visit, and capture rate according to location. Table 2 shows bats morphometric measurements that are translated into weight to length (W-L) percentages, and bats conservation status in the wild that were acquired from International Union for Conservation of Nature (IUCN) Red List of Threatened Species [1]. Table 2 also includes bats common name and local name, allometric description, and their guild type. Table 3 displays complete raw data on bats capture along with additional morphological descriptions, sex, life stage, and trapping method.

**Table 1:** Taxonomic classification and abundance of bats abundance discovered from the study sites within districts Setiu and Hulu Terengganu.

FAMILY	SPECIES	SETIU		HULU TERENGGANU		N	Relative abundance (%)
		A	B	C	D		
Pteropodidae	*Cynopterus brachyotis*	4	8	1	0	13	16.9
	*Cynopterus horsfieldii*	2	0	1	0	3	3.9
	*Balionycteris maculata*	2	1	2	1	6	7.8
	*Penthetor lucasi*	0	0	0	1	1	1.3
Hipposideridae	*Hipposideros ater*	0	0	2	9	11	14.3
	*Hipposideros bicolor*	0	0	9	19	28	36.4
	*Hipposideros galeritus*	0	0	0	3	3	3.9
	*Hipposideros doriae*	0	0	0	2	2	2.6
	*Hipposideros cineraceus*	0	0	0	1	1	1.3
	*Hipposideros larvatus*	0	0	0	2	2	2.6
	*Hipposideros cervinus*	0	0	1	0	1	1.3
Rhinolophidae	*Rhinolophus convexus*	0	1	0	3	4	5.2
	*Rhinolophus affinis*	0	1	0	0	1	1.3
						0	
Vespertilionidae	*Kerivoula pellucida*	0	0	1	0	1	1.3
	*Murina suilla*	0	0	1	0	1	1.3
Abundance		8	11	18	41	78	100
Species (No.)		3	4	8	9	15	
Field visits (Days)		6	6	6	6	24	
Capture rate (%)		13.3	18.3	30.0	68.3	32.5	

*Note:* The sites are described as A = Ladang Tayor TDM, B = Bukit Kesing Forest Reserve, C = Pengkalan Utama, Kenyir and D = Sungai Buweh, Kenyir. Annotation ‘N’ represents number of bats.

**Table 2:** Identity, statuses in the wild, length to weight percentage, allometric description and guild for bats captured from the study sites within districts Setiu and Hulu Terengganu.

Species	Common name	Local name	Status	W/L (%)	Description	Guild
Bukit Kesing Forest Reserve, Setiu						
*Cynopterus brachyotis*	Lesser short-nosed fruit bat	Cecadu pisang	LC	44.0 ± 1.6 ^(N = 8)^	NA	Frugivorous bat
*Balionycteris maculata*	Spotted-winged fruit bat	Cecadu sayap bertitik	LC	32.3 ± 0.0 ^(N = 1)^	NA	Insectivorous bat
*Rhinolophus convexus*	Convex horseshoe bat	-	DD	17.6 ± 0.0 ^(N = 1)^	NA	Insectivorous bat
*Rhinolophus affinis*	Intermediate horseshoe bat	Kelawar ladam hutan	LC	29.0 ± 0.0 ^(N=1)^	NA	Insectivorous bat
Ladang Tayor TDM, Setiu						
*Cynopterus brachyotis*	Lesser short-nosed fruit bat	Cecadu pisang	LC	45.1 ± 2.4 ^(N = 4)^	NA	Frugivorous bat
*Cynopterus horsfieldii*	Horsfield's fruit bat	Cecadu pisang besar	LC	69.4 ± 0.0 ^(N = 1)^	PA	Insectivorous bat
*Balionycteris maculata*	Spotted-winged fruit bat	Cecadu sayap bertitik	LC	27.2 ± 3.7 ^(N = 2)^	NA	Frugivorous bat
Pengkalan Utama Kenyir, Hulu Terengganu						
*Cynopterus brachyotis*	Lesser short-nosed fruit bat	Cecadu pisang	LC	45.2 ± 0.0 ^(N = 1)^	NA	Frugivorous bat
*Cynopterus horsfieldii*	Horsfield's fruit bat	Cecadu pisang besar	LC	71.9 ± 0.0 ^(N = 1)^	PA	Frugivorous bat
*Balionycteris maculata*	Spotted-winged fruit bat	Cecadu sayap bertitik	LC	32.5 ± 1.8 ^(N = 2)^	NA	Frugivorous bat
*Hipposideros ater*	Dusky leaf-nosed bat	Kelawar ladam bulat biasa	LC	14.2 ± 0.7 ^(N = 2)^	NA	Insectivorous bat
*Hipposideros bicolor*	Bicolored leaf-nosed bat	Kelawar ladam bulat biasa	LC	15.8 ± 0.9 ^(N = 9)^	NA	Insectivorous bat
*Hipposideros cervinus*	Fawn-colored leaf-nosed bat	Kelawar ladam bulat gua	LC	20.4 ± 0.0 ^(N = 1)^	NA	Insectivorous bat
*Kerivoula pellucida*	Clear-winged woolly bat	Kelawar kepak jernih	NT	14.2 ± 0.0 ^(N = 1)^	NA	Insectivorous bat
*Murina suilla*	Brown tube-nosed bat	Kelawar hidung laras kecil	LC	11.0 ± 0.0 ^(N = 1)^	NA	Insectivorous bat
Sungai Buweh, Hulu Terengganu						
*Balionycteris maculata*	Spotted-winged fruit bat	Cecadu sayap bertitik	LC	26.0 ± 0.0 ^(N = 2)^	NA	Frugivorous bat
*Penthetor lucasi*	Lucas's short-nosed fruit Bat	Cecadu hitam-pudar	LC	62.3 ± 0.0 ^(N = 1)^	PA	Frugivorous bat
*Hipposideros ater*	Dusky leaf-nosed bat	Kelawar ladam hitam-pudar	LC	15.7 ± 0.7 ^(N = 9)^	NA	Insectivorous bat
*Hipposideros bicolor*	Bicolored leaf-nosed bat	Kelawar ladam bulat biasa	LC	15.3 ± 0.3 ^(N = 19)^	NA	Insectivorous bat
*Hipposideros galeritus*	Cantor's leaf-nosed bat	Kelawar ladam cantor	LC	14.4 ± 0.3 ^(N = 3)^	NA	Insectivorous bat
*Hipposideros doriae*	Bornean leaf-nosed bat	Kelawar ladam bulat lawas	NT	10.3 ± 1.1 ^(N = 2)^	NA	Insectivorous bat
*Hipposideros cineraceus*	Ashy roundleaf bat	Kelawar ladam bulat terkecil	LC	17.7 ± 0.0 ^(N = 1)^	NA	Insectivorous bat
*Hipposideros larvatus*	Intermediate roundleaf bat	Kelawar ladam bulat besar	LC	29.4 ± 1.5 ^(N = 2)^	NA	Insectivorous bat
*Rhinolophus convexus*	Convex horseshoe bat	-	DD	18.7 ± 4.2 ^(N = 3)^	NA	Insectivorous bat

*Note:* Identity and statuses of bats follow IUCN Red List descriptions whereby LC = Least Concern, NT = Near Threatened, and DD = Data Deficient. The Weight-Length ratio represented as W/L are measured using division of weight against total length of animal and measured as percentage (%). The annotations in brackets, ‘N’ represents number of animals handled to obtain the desired measurements. Additionally, the Weight to Length (W/L) percentages are described as quartiles represented by < 50 % = negative allometric [NA] (Size exceeds body weight), 50 % = symmetric (Body weight increases with size) and > 50% = positive allometric [PA] (Body weight exceeds size).

**Table 3:** The unprocessed data of bats captured from study sites within districts Setiu and Hulu Terengganu.

Num.	Date	Species	Trap	Sex	Stage	TL (mm)	E (mm)	TB (mm)	HF (mm)	T (mm)	WT (g)
Bukit Kesing Forest Reserve, Setiu											
1	25 October, 2018	*Balionycteris maculata*	MN	F	J	47	11	18	8	9	12.8
2	26 October, 2018	*Cynopterus brachyotis*	MN	M	A	60	14	22	13	12	28.8
3	26 October, 2018	*Cynopterus horsefieldii*	MN	F	A	75	20	28	14	18	47.9
4	26 October, 2018	*Cynopterus brachyotis*	MN	F	J	63	18	24	12	12	24.5
5	16 November, 2018	*Balionycteris maculata*	MN	F	J	44	10	15	6	3	11.9
6	16 November, 2018	*Cynopterus horsefieldii*	MN	F	A	71	21	29	16	18	49.3
7	16 November, 2018	*Cynopterus brachyotis*	MN	M	A	64	16	24	10	11	31.7
8	17 November, 2018	*Cynopterus brachyotis*	MN	F	A	64	19	21	11	11	28.2
Ladang Tayor TDM, Setiu											
1	27 October, 2018	*Cynopterus brachyotis*	MN	M	A	62	17	22	10	10	26.9
2	28 October, 2018	*Rhinolopus convecus*	HT	F	J	41	16	17	5	22	7.2
3	30 November, 2018	*Cynopterus brachyotis*	HT	M	A	65	16	25	25	12	30.2
4	30 November, 2018	*Balionycteris maculata*	MN	F	A	40	8	17	17	10	12.9
5	30 November, 2018	*Cynopterus brachyotis*	MN	M	A	62	18	29	29	7	31.0
6	1 December, 2018	*Cynopterus brachyotis*	MN	F	J	61	14	21	12	15	27.7
7	1 December, 2018	*Cynopterus brachyotis*	MN	M	A	65	21	22	11	17	27.4
8	1 December, 2018	*Cynopterus brachyotis*	MN	M	A	64	19	22	11	11	26.1
9	1 December, 2018	*Cynopterus brachyotis*	MN	M	A	65	17	22	11	14	23.4
10	1 December, 2018	*Cynopterus brachyotis*	MN	F	A	60	19	22	13	19	28.9
11	16 January, 2019	*Rhinolopus affinis*	MN	M	A	52	17	24	9	26	15.1
Pengkalan Utama, Kenyir, Hulu Terengganu											
1	2 January, 2019	*Murina suilla*	HT	M	A	30	12	15	6	32	3.3
2	3 January, 2019	*Balionycteris maculata*	MN	F	J	44	10	12	7	12	15.1
3	3 January, 2019	*Balionycteris maculata*	MN	F	A	48	13	16	8	5	14.7
4	3 January, 2019	*Cynopterus horsefieldii*	MN	M	A	74	18	29	10	13	53.2
5	3 January, 2019	*Hipposideros bicolor*	HT	F	A	44	14	19	7	22	6.3
6	3 January, 2019	*Hipposideros bicolor*	HT	F	A	44	16	21	6	23	6.2
7	5 January, 2019	*Hipposideros bicolor*	HT	M	A	44	15	17	6	21	7.6
8	5 January, 2019	*Hipposideros bicolor*	HT	F	A	44	16	17	6	22	6.7
9	5 January, 2019	*Hipposideros bicolor*	HT	F	A	45	15	17	7	20	7.6
10	5 January, 2019	*Kerivola pelucida*	HT	F	A	31	13	17	8	45	4.4
11	5 January, 2019	*Hipposideros bicolor*	HT	F	A	44	17	16	6	26	6.9
12	5 January, 2019	*Hipposideros ater*	HT	F	A	42	15	17	6	22	5.7
13	5 January, 2019	*Hipposideros bicolor*	HT	F	A	36	13	15	5	24	7.8
14	5 January, 2019	*Hipposideros bicolor*	HT	M	A	45	16	18	6	23	5.5
15	6 January, 2019	*Hipposideros cervicus*	HT	M	A	49	18	19	7	23	10.0
16	6 January, 2019	*Hipposideros ater*	HT	M	A	43	16	17	7	21	6.4
17	6 January, 2019	*Hipposideros bicolor*	HT	M	A	44	16	17	6	24	6.6
18	6 January, 2019	*Cynopterus brachyotis*	MN	M	J	60	17	22	11	11	27.1
Sungai Buweh, Kenyir, Hulu Terengganu											
1	3 January, 2019	*Balionycteris maculata*	MN	F	J	42	9	16	7	5	10.9
2	3 January, 2019	*Hipposideros ater*	HT	M	A	43	14	19	7	36	7.6
3	4 January, 2019	*Hipposideros bicolor*	HT	F	A	44	15	18	7	24	6.5
4	4 January, 2019	*Hipposideros bicolor*	HT	M	A	44	12	17	6	26	6.3
5	4 January, 2019	*Hipposideros bicolor*	HT	F	A	44	17	17	5	23	6.1
6	4 January, 2019	*Hipposideros bicolor*	HT	M	A	44	17	17	5	24	6.8
7	4 January, 2019	*Hipposideros bicolor*	HT	F	A	44	16	16	5	22	6.1
8	4 January, 2019	*Hipposideros bicolor*	HT	M	A	45	17	17	5	25	6.9
9	4 January, 2019	*Hipposideros bicolor*	HT	F	A	45	19	17	5	24	6.3
10	4 January, 2019	*Hipposideros bicolor*	HT	F	A	44	17	17	5	24	6.5
11	4 January, 2019	*Hipposideros ater*	HT	M	A	43	17	17	6	23	6.5
12	4 January, 2019	*Hipposideros ater*	HT	F	A	43	16	17	4	22	6.3
13	4 January, 2019	*Hipposideros galentus*	HT	M	A	49	15	22	7	40	7.3
14	4 January, 2019	*Hipposideros larvatus*	HT	M	A	60	17	24	9	30	18.5
15	4 January, 2019	*Hipposideros doriae*	HT	M	A	37	15	17	5	26	3.4
16	5 January, 2019	*Hipposideros ater*	HT	M	A	43	16	18	6	23	6.3
17	5 January, 2019	*Hipposideros ater*	HT	M	A	43	17	17	6	22	6.3
18	5 January, 2019	*Hipposideros ater*	HT	M	A	43	14	18	6	21	6.6
19	5 January, 2019	*Hipposideros ater*	HT	M	A	43	16	19	7	25	8.7
20	5 January, 2019	*Hipposideros ater*	HT	M	A	43	16	17	7	20	6.2
21	5 January, 2019	*Hipposideros ater*	HT	M	A	43	16	18	6	23	6.3
22	5 January, 2019	*Hippposideros bicolor*	HT	M	A	46	15	17	6	27	7.0
23	5 January, 2019	*Hippposideros bicolor*	HT	F	A	46	16	17	6	21	6.8
24	5 January, 2019	*Hippposideros bicolor*	HT	F	A	47	16	17	6	27	6.9
25	5 January, 2019	*Hippposideros bicolor*	HT	M	A	45	16	17	6	26	6.5
26	5 January, 2019	*Hippposideros bicolor*	HT	M	A	45	16	20	8	27	8.0
27	5 January, 2019	*Hippposideros bicolor*	HT	M	A	44	14	18	7	22	6.4
28	5 January, 2019	*Hippposideros bicolor*	HT	F	A	44	15	19	7	32	7.2
29	5 January, 2019	*Hippposideros bicolor*	HT	F	A	47	17	18	6	23	7.9
30	5 January, 2019	*Hippposideros bicolor*	HT	M	A	45	16	18	6	24	7.1
31	5 January, 2019	*Hippposideros bicolor*	HT	M	A	45	16	18	6	22	8.2
32	5 January, 2019	*Hippposideros bicolor*	HT	M	A	46	17	18	5	22	6.8
33	5 January, 2019	*Hipposideros galentus*	HT	M	A	46	11	20	5	39	6.5
34	5 January, 2019	*Hipposideros galentus*	HT	F	A	47	14	22	7	46	6.6
35	5 January, 2019	*Hipposideros doriae*	HT	F	J	37	16	14	7	25	4.2
36	5 January, 2019	*Hipposideros cinerateus*	HT	M	A	47	16	21	7	34	8.3
37	5 January, 2019	*Hipposideros larvatus*	HT	M	A	58	15	24	11	30	16.2
38	5 January, 2019	*Rhinolopus convecus*	HT	M	J	40	15	16	8	24	10.8
39	5 January, 2019	*Rhinolopus convecus*	HT	F	A	43	16	16	7	22	6.0
40	6 January, 2019	*Rhinolopus convecus*	HT	M	A	41	14	17	9	22	6.2
41	6 January, 2019	*Penthetor lucasi*	MN	M	A	64	17	28	12	11	39.9

*Note:* Bats counts are represented by (num.), traps used are denote with HT = harp trap and MN = mist net, sex are denote with M = male and F = female, and life stage are denote with A = adult and J = juvenile. Description of measurements are abbreviated as TL = total length (from nose tip to end of tail), E = ear length, TB = tibia length, HF = hind foot length, T = tail length and WT = weight. Measurements are denote with g = gram and mm = millimeter.

## 2 Experimental Design, Materials, and Methods

Our field team visited two districts Setiu (Bukit Kesing Forest Reserve and Ladang Tayor TDM) and Hulu Terengganu (Pengkalan Utama and Sungai Buweh, Kenyir) between October 2018 and January 2019. We sampled each site for seven days (six nights). We used four-bank harp traps, which we set up about 1 m above the ground level and mist nets with the help of two poles to support the net following [2]. Hourly every day after sunset and before sunrise (between 1830 and 0630), we extracted captured bats that were entangled in the nests or in the collecting bag of the harp trap . We safely secured them in cloth bags before the sex, life stage, and external measurement of the bats could be recorded by measuring the forearm, tail, tibia, hind foot, and ear length using Vernier caliper. We recorded the mass of each bat using a portable analytical balance (sensitivity ± 0.01 g) following [3]. To construct the Body Condition Index adopted from [4], we used the descriptive measurements of each bat such as forearm length and mass. In the presence of negative values, the body mass scale was adapted with the values and separated by 0.5 differential margins that give rise to underweight, ideal, overweight and obese. We identified the bats up to species level using an identification key following [5]. At the end of the data collection we released the bats back into the wild at the capture site.

## Appendix Supplementary materials

Image, application 1
